# Supplementary material for: Discovery of Novel TASK-3 Channel Blockers Using a Pharmacophore-Based Virtual Screening
Source: Int J Mol Sci. 2019 Aug 17;20(16):4014. doi: 10.3390/ijms20164014 (PMC6720600; doi:10.3390/ijms20164014)
Supplement: Supplementary file 1 [file ijms-20-04014-s001.pdf]

Discovery of novel TASK-3 channel blockers using a pharmacophore-based virtual screening.

David Ramírez, Guierdy Concha, Bárbara Arévalo, Luis Prent-Peñaloza, Leandro Zúñiga, Aytug K. Kiper, Susanne Rinné, Miguel Reyes-Parada, Niels Decher, Wendy González, Julio Caballero.

Table S1. e-Pharmacophore general site measurements. *e*-Pharmacophore hypothesis No.1 site measurements distances(Å) and angles (degrees).

Site 1	Site 2		Distance
A1	*A2*		5.943
A1	*R*		8.366
A2	*R*		3.654
**Site 1**	**Site 2**	**Site 3**	**Angle**
A1	*A2*	*R*	119.4
A1	*R*	*A2*	38.2
A2	*A1*	*R*	22.4

A1, A2 = Hydrogen bond acceptors, *R* = aromatic ring.

Table S2. Binding site residues in the TASK-3 model T3-twiOO of DR16 and DR16.1. *First column*, residues interacting only with DR16. *Second column*, residues interacting with DR16 and DR16.1. *Third column*, residues interacting only with DR16.1. Residues were collected from the 2D diagrams (Figures 3D and 4B). These diagrams show residues within 5 Angstrom of the drugs.

DR16	Common residues	DR16.1
Val115	Thr198	Asn133
Leu171	Leu197	Thr121
Phe194	Thr199	Phe125
	Thr233	Thr93*
	Leu232	Thr92
	Leu239*	Gln126*
	Ile235	Glu130
	Gly236*	Ala237*
	Leu122 (Chain A)*	Leu122 (Chain B)*
	Pro119	Gly129
	Ile118	Asn240
Yellow	Residues of the A1899 binding site	
*	Residues of the PK-THPP binding site	

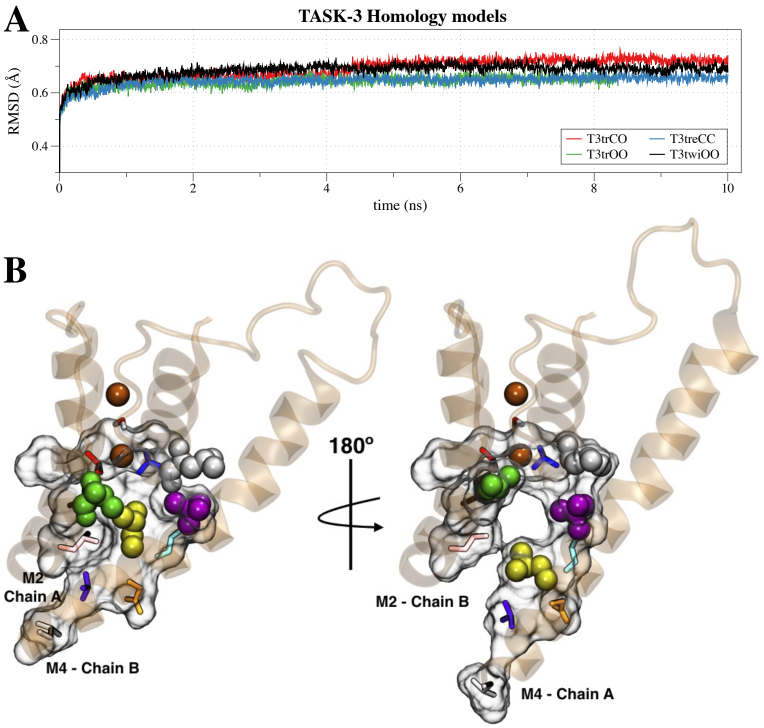

Figure S1. A. RMSD for backbone atoms of TASK-3 homology models during the 10ns–MDs. RMSD for T3trCO, T3trOO, T3treCC and T3twiOO are represented in red, green, blue and black respectively. **B.** Fenestration states representation in T3-trCO model. Left. Close fenestration state. Right. Open fenestration state. Residues Thr93 (red) of the first pore loop (P1), Thr199 (blue) of the second pore loop (P2), Ile118 (black) and Leu122 (pink) of the M2 segment, Gly236 (cyan), Asn240 (orange), Val243 (violet) and Leu247 (silver) of the M4 segment are shown in sticks representation. Val 115, (green), Leu197 (white), Ile235 (magenta) and Leu239 (yellow) are showed in van der Waals representation. T3-trCO (orange) is showed in cartoon representation. For better display of fenestration state, the T3-trCO model is shown in two orientations (rotated by 180°). K+ ions located in S2 and S4 positions are showed in van der Waals representation. Waters located in S3 position is showed in sticks representation.

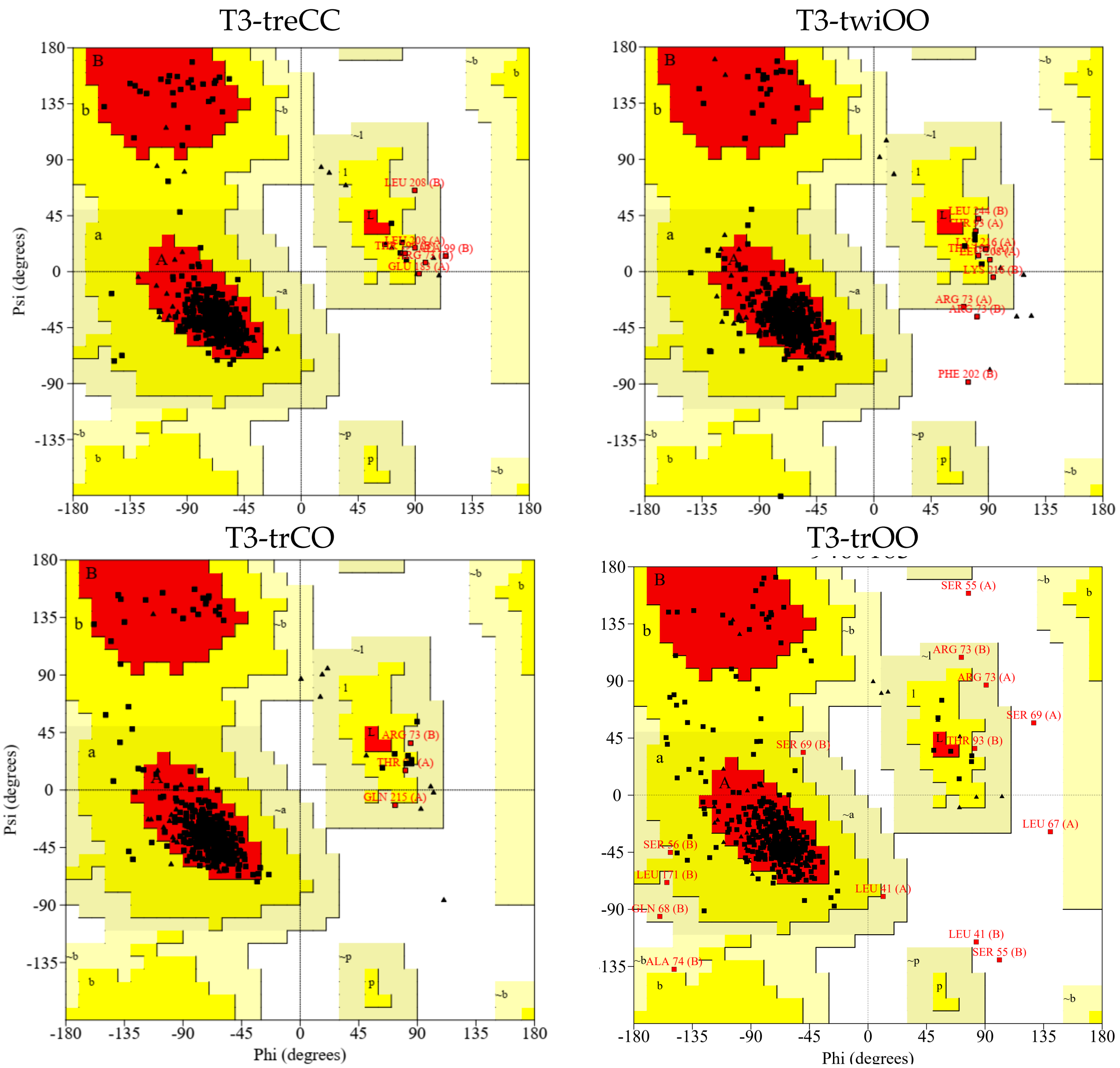

Model		Residues in most favored regions (%)	Residues in disallowed regions (%)
**A**	T3-treCC	94.3	0.2
**B**	T3-twiOO	91.1	0.5
**C**	T3-trCO	93.5	0.0
**D**	T3-trOO	90.2	0.4

Figure S2. Procheck validation of the homology models. Ramachandran plot and statistics of TASK-3 homology models.

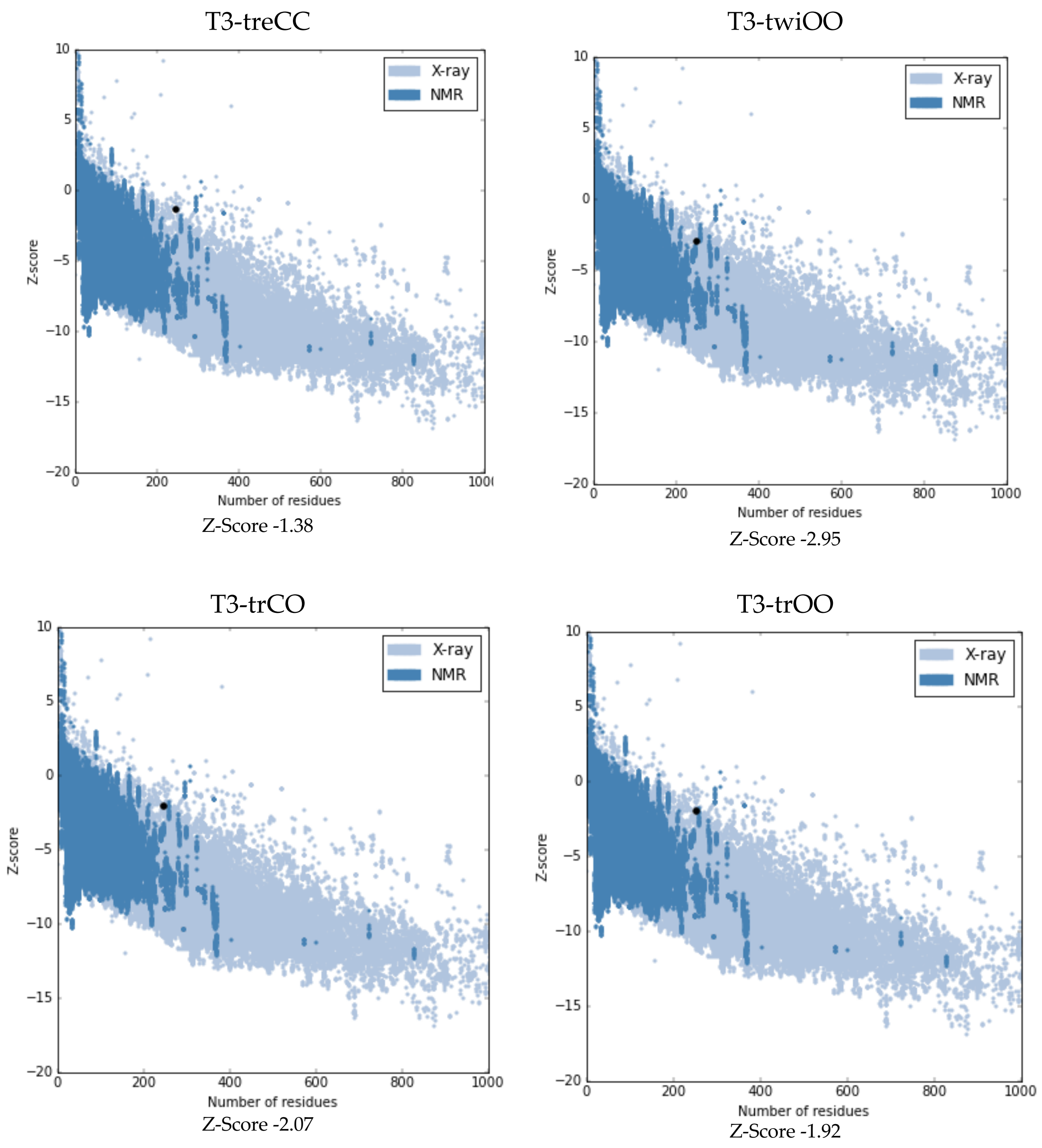

Figure S3. ProSA validation. Z-Score plots comparing the TASK-3 homology models with the structures in the Protein DataBank.

DR1

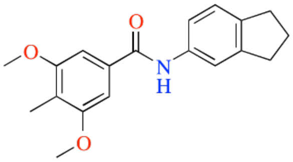

DR2

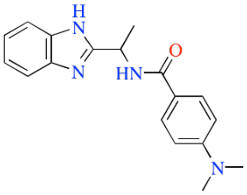

DR3

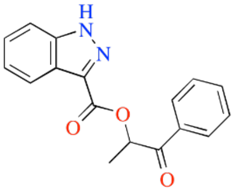

DR4

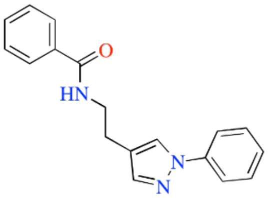

DR5

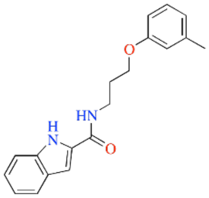

DR6

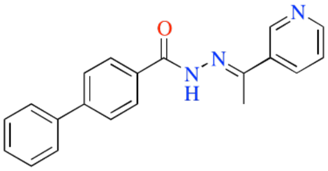

DR7

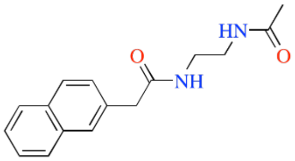

DR8

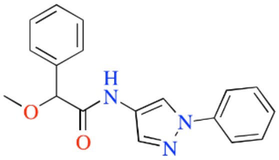

DR9

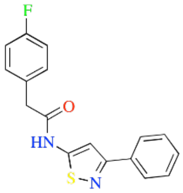

DR10

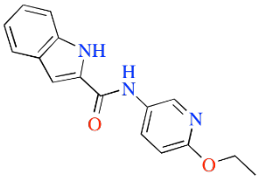

DR11

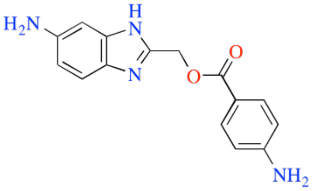

DR12

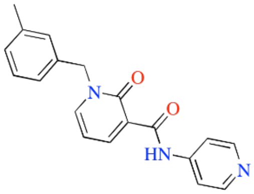

DR13

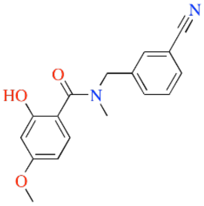

DR14

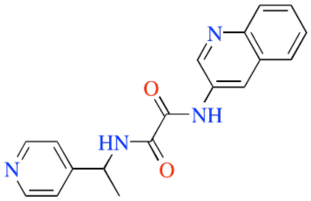

DR15

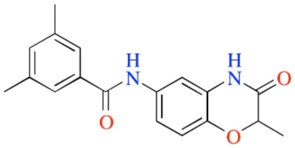

DR16

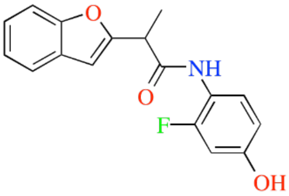

DR17

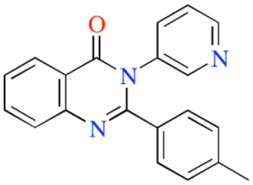

DR18

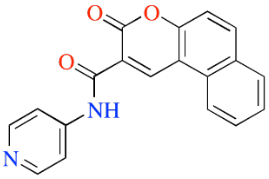

DR19

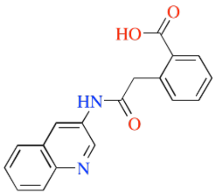

Figure S4. Identified hits through virtual screening protocol.

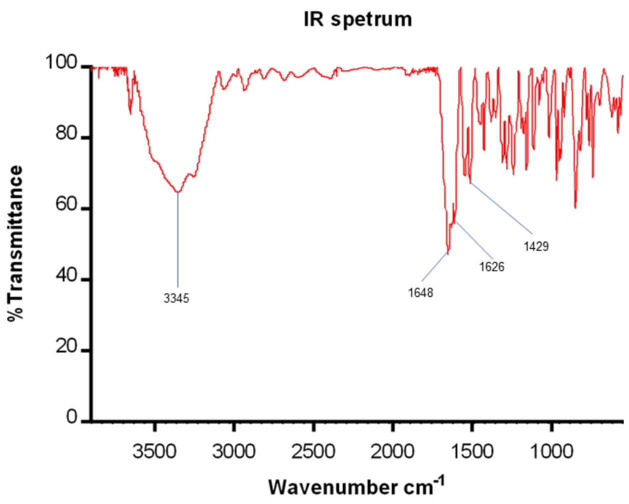

Figure S5. FT-IR spectrum of DR16.1.

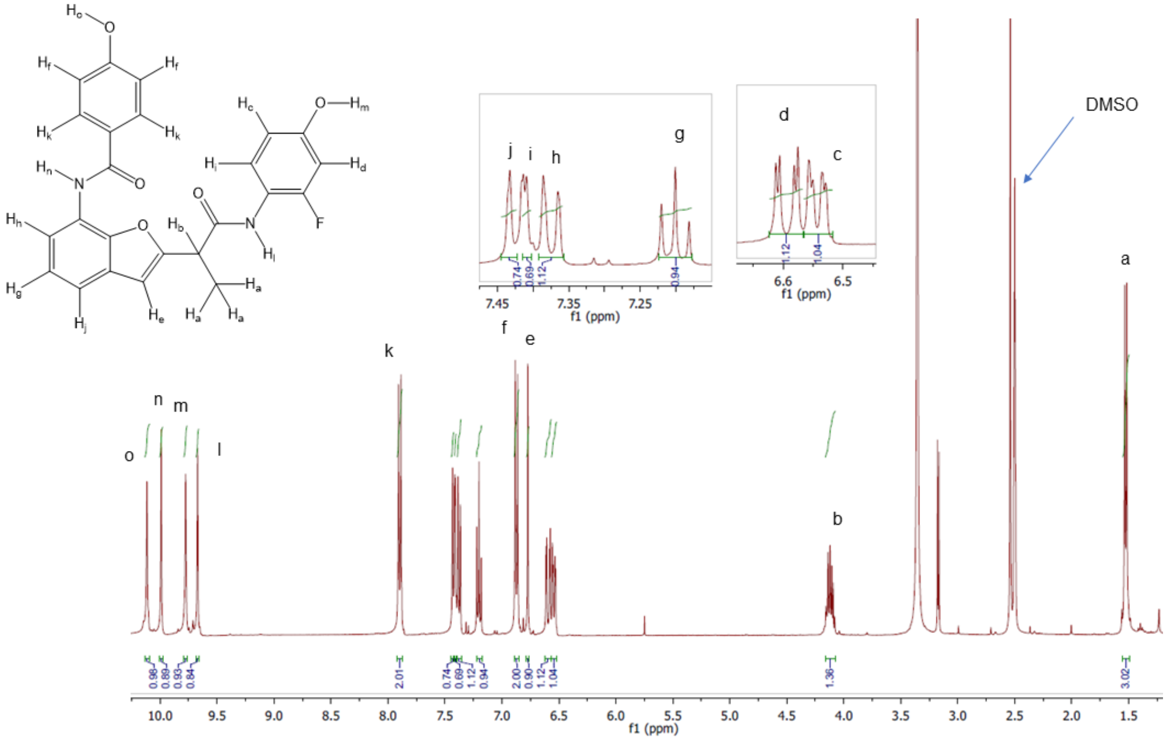

Figure S6. 400 MHz 1H-NMR spectrum in DMSO- d6 of DR16.1.

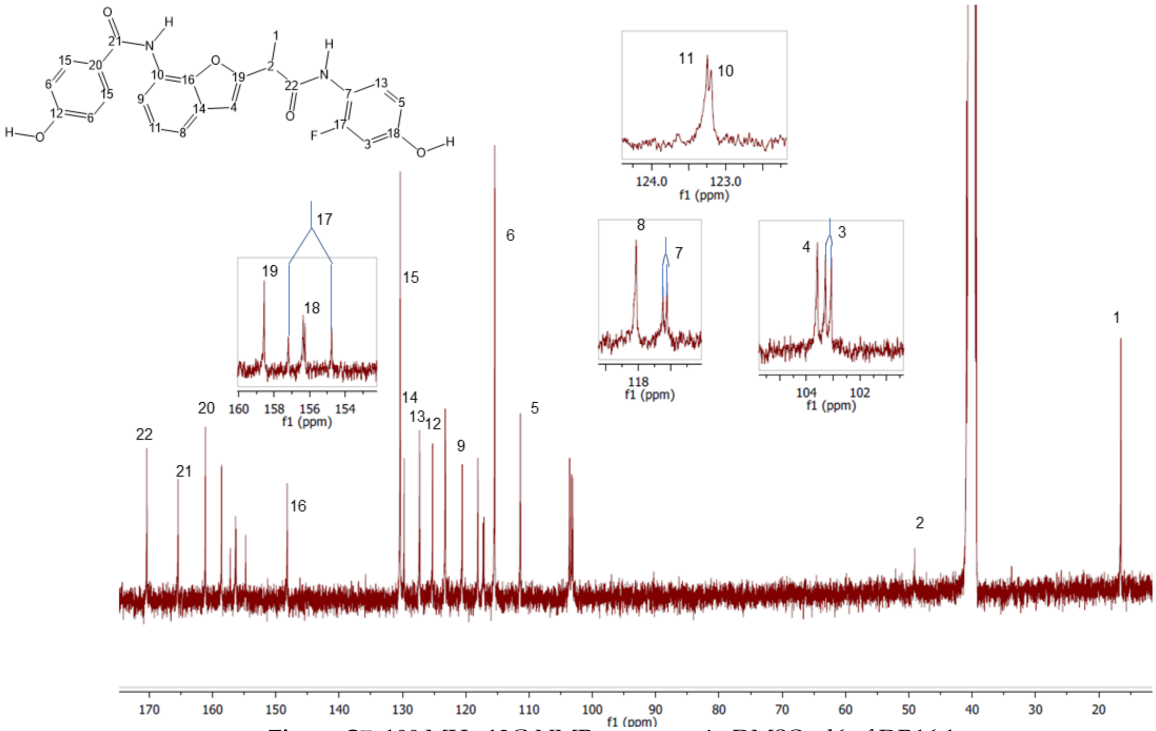

Figure S7. 100 MHz 13C-NMR spectrum in DMSO- d6 of DR16.1.

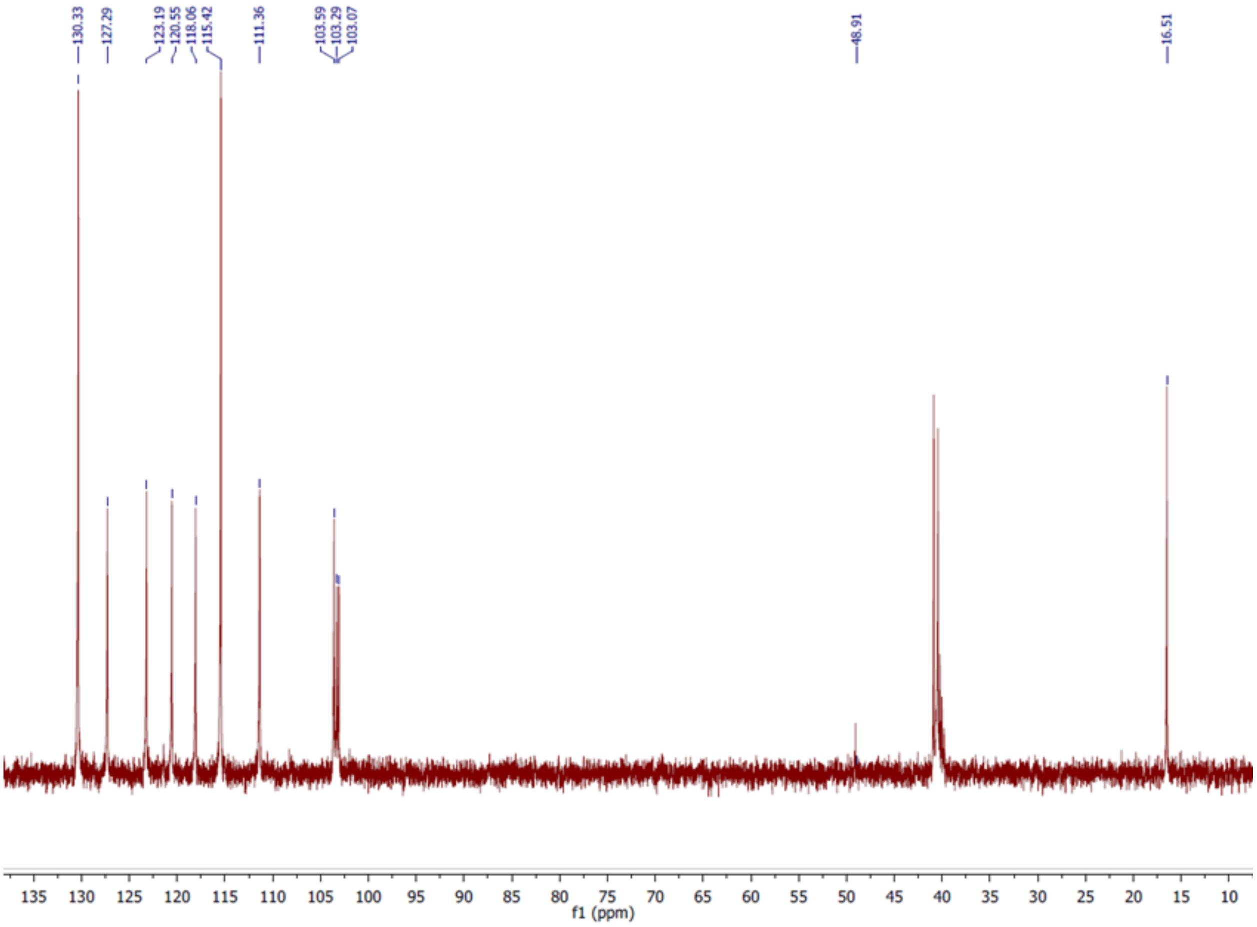

Figure S8. DEPT-135 spectrum in DMSO- d6 of DR16.1.

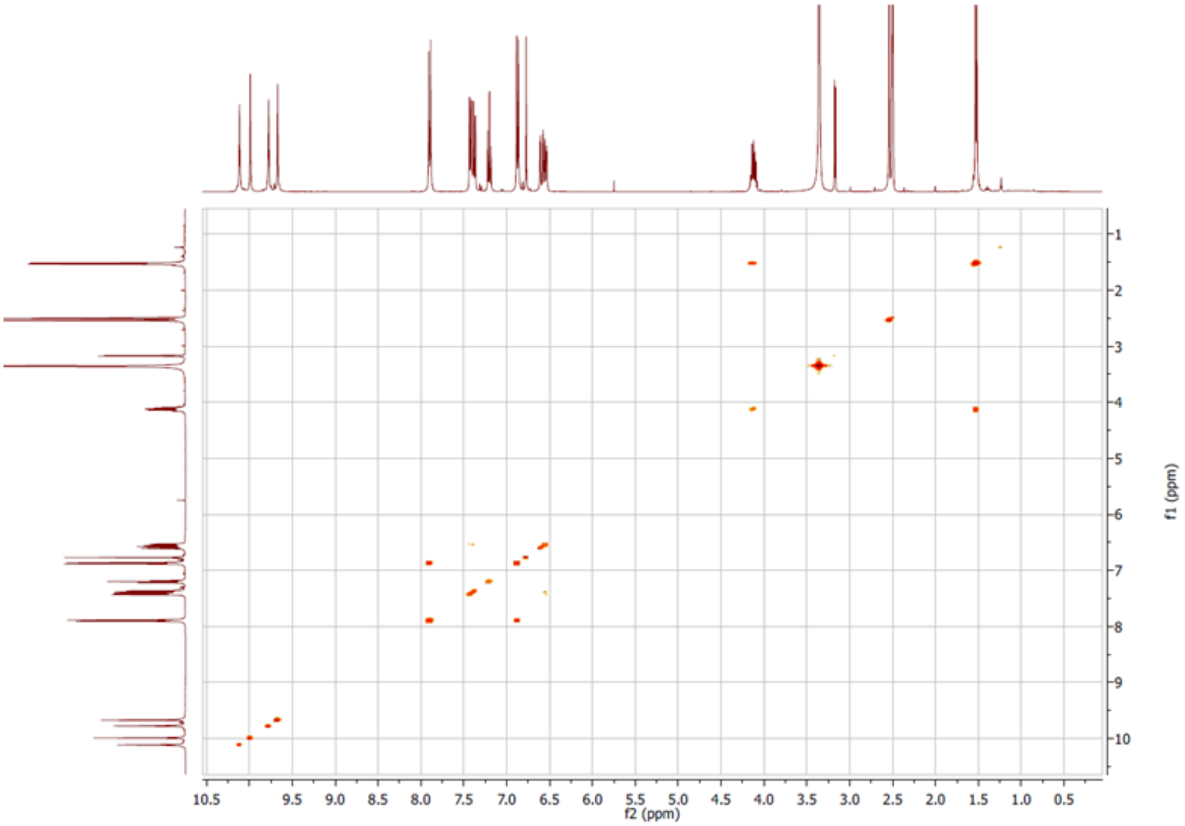

Figure S9. COSY spectrum in DMSO- d6 of DR16.1.

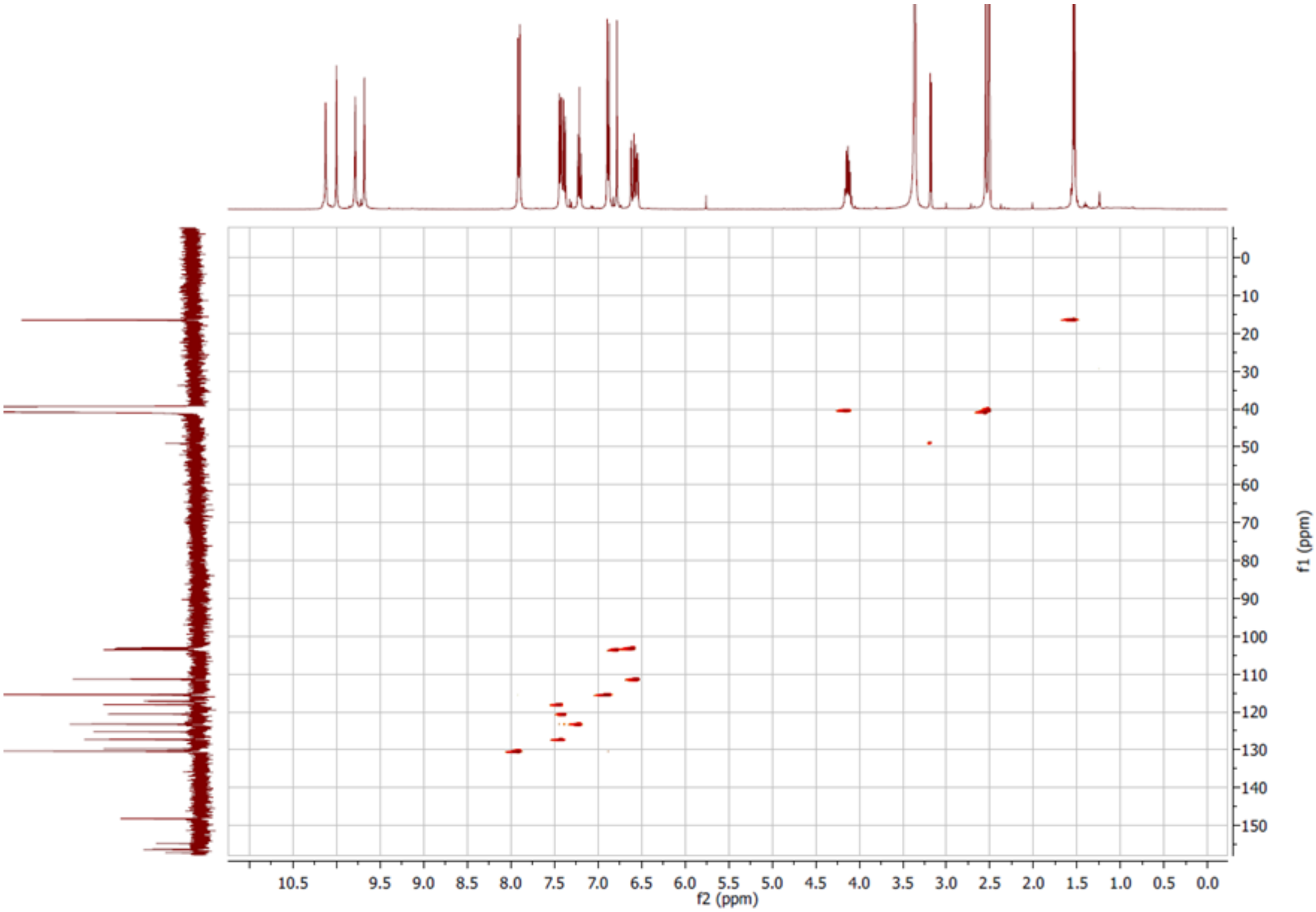

Figure S10. HMQC spectrum in DMSO- d6 of DR16.1.

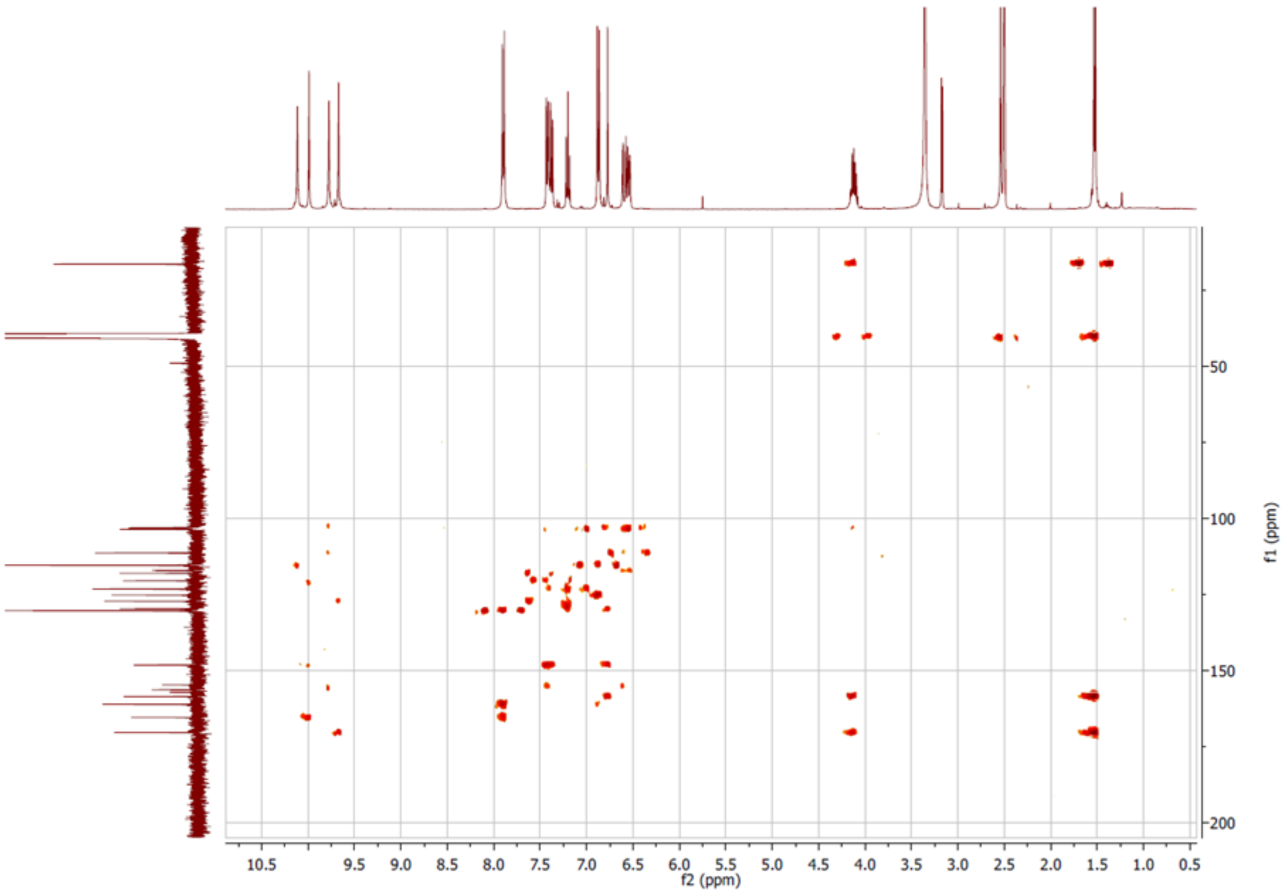

Figure S11. HMBC spectrum in DMSO- d6 of DR16.1.

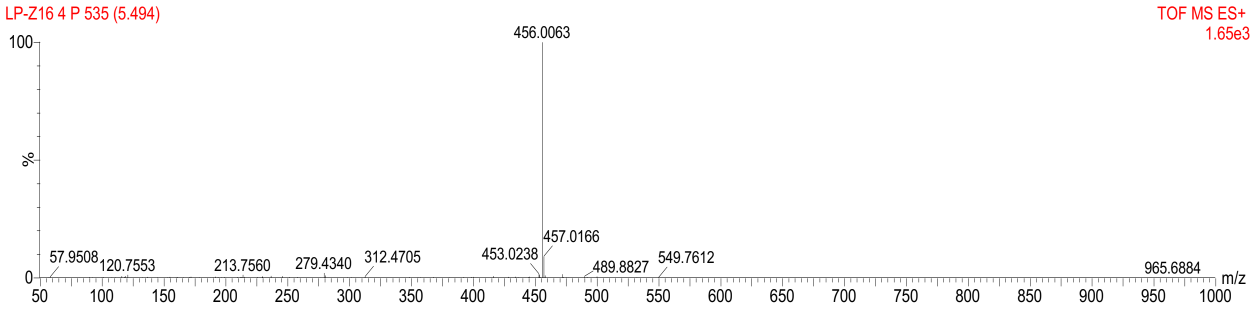

Figure S12. Mass spectrum of DR16.1 with positive ionization.

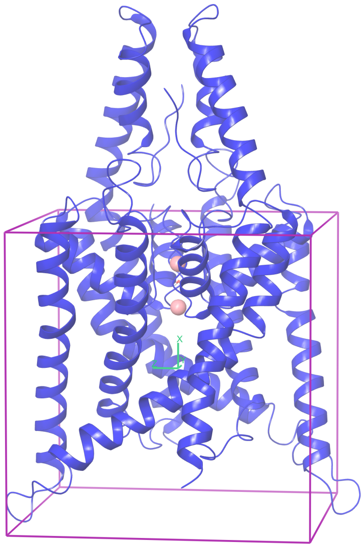

Figure S13. Grid box of the T3-twiOO model (blue) used in the study. The dimensions of the grid box were (35 × 35 × 35) $Å^3$. The same grid box was used for all TASK-3 models.
